# A consumer-targeted, pharmacist-led, educational intervention to reduce inappropriate medication use in community older adults (D-PRESCRIBE trial): study protocol for a cluster randomized controlled trial

**DOI:** 10.1186/s13063-015-0791-1

**Published:** 2015-06-10

**Authors:** Philippe Martin, Robyn Tamblyn, Sara Ahmed, Andrea Benedetti, Cara Tannenbaum

**Affiliations:** Institut Universitaire de Gériatrie de Montréal, Faculté de Pharmacie, Université de Montréal, Montréal, QC Canada; Department of Epidemiology, Biostatistics and Occupational Health, McGill University, Montreal, QC Canada; School of Physical and Occupational Therapy, McGill University, Montreal, QC Canada

**Keywords:** Patient education, Inappropriate prescription, Pharmaceutical opinion, Cognition disorders/drug therapy, Polypharmacy

## Abstract

**Background:**

Medication safety for older persons represents an ongoing challenge. Inappropriate prescriptions – those with a high risk of evidence-based harm – persist in up to 25 % of seniors, and account for a significant proportion of avoidable emergency department visits. This project is the sequel to the EMPOWER study, in which a novel consumer-targeted written knowledge transfer tool aimed at empowering older adults to act as drivers of benzodiazepine de-prescription resulted in a 27 % reduction of inappropriate benzodiazepine use at 6-month follow-up (number needed to treat (NNT) = 4). Failure to discontinue in the EMPOWER study was attributable to re-emerging symptoms among participants, prescribing inertia, and lack of knowledge and skills for substituting alternate therapy among physicians and pharmacists. To maximize de-prescription of inappropriate therapy, educational medication-risk reduction initiatives should be tested that simultaneously include patients, physicians and pharmacists. The objective of this trial is to: 1) test the beneficial effect of a new de-prescribing paradigm enlisting pharmacists to transfer knowledge to both patients and prescribers in a 2-pronged approach to reduce inappropriate prescriptions, compared to usual care and 2) evaluate the transferability of the EMPOWER study concept to other classes of inappropriate prescriptions.

**Methods:**

We intend to conduct a 3-year pragmatic cluster randomized parallel-group controlled trial to test the effect of the new de-prescribing intervention compared to usual care for reducing 4 classes of inappropriate prescriptions from the 2012 Beers criteria among 450 community-dwelling older adults with polypharmacy. Inappropriate prescriptions will include benzodiazepines, sulfonylurea hypoglycemic agents, first generation antihistamines and non-steroidal anti-inflammatory drugs. The study population is community-dwelling older adults recruited from community pharmacies in Quebec, Canada. The intervention was developed based on a systematic review of the evidence for each medication. Participants in the experimental group will receive the written educational program following randomization and have their pharmacist send their physicians an evidence-based pharmaceutical opinion to recommend de-prescription and be followed for a year. The control group will be wait-listed for 6 months.

**Discussion:**

System change to effectively reduce medication risk among community-dwelling seniors requires a coordinated approach targeting physicians, pharmacists and patients. This trial will test the feasibility and effectiveness of a tripartite approach to de-prescribing.

**Trial registration:**

Registered via ClinicalTrials.gov on 31 January 2014, identifier: NCT02053194.

## Background

Older adults rank concerns about medication side effects highest on their list of health priorities, with 89 % of those with chronic conditions willing to attempt cessation of one of their medications if deemed appropriate by a physician [1–3]. Seniors have good reason to be concerned: as life expectancy improves and older adults live longer with chronic conditions, they are also more likely to consume multiple medications [4, 5]. Polypharmacy is a risk factor for adverse drug events including drug-drug interactions, emergency department visits due to therapeutic competition, hospitalization and death [6–8]. Some medications confer greater risk than others, and are termed inappropriate when their risks outweigh the benefits, and when safer therapeutic alternatives exist that have similar or superior efficacy [9–11].

Despite the development of guidelines identifying inappropriate medications among older adults such as the Beers criteria [9], inappropriate prescriptions persist in up to 25 % of community-dwelling non-hospitalized older adults aged 65+, depending on the criteria used and the country studied [10, 12]. Interventions aimed at physicians and pharmacists for reducing inappropriate medication use include medication reviews and software alerts [13, 14]. In a previous study [15], we developed and tested a consumer-targeted written knowledge transfer tool aimed at empowering older adults to act as drivers of safer prescribing practices. This resulted in a 27 % discontinuation rate in the intervention group independent of patient factors [15] and thus EMPOWER provided proof of concept that directly targeting consumers as drivers of safer prescriptions can be effective for reducing medication risk.

Several challenges and opportunities became apparent in the EMPOWER study. Patients stated in 33 % of cases that physicians were reluctant to change their prescription. Second, we realized that if the de-prescribing process were to become sustainable over the longterm, the new paradigm would have to be entrenched within the pharmaceutical sector and involve the prescriber, the patient and the pharmacist.

A tripartite approach to de-prescribing is supported by a recent systematic review on the barriers of de-prescribing, which suggests that the decision to stop a medication by an individual is influenced by multiple competing barriers and enablers [16]. In this review, a total of four enablers and barriers to de-prescribing were identified. Enablers consisted of agreement with appropriateness of cessation, positive influences such as support from the pharmacist and/or physician, dislike of medication as well as the presence of a clear cessation process. Barriers to cessation consisted of fear of cessation, negative influences such as discouragement from the pharmacist and/or physician, disagreement over the appropriateness of cessation, as well as the absence of a clear cessation process. Using this knowledge as well as our own findings from the EMPOWER study, which also demonstrated barriers to cessation such as prescribing inertia and a lack of knowledge and skills for substituting alternate therapy, we developed the current approach to the patient de-prescribing process. This trial aims to address these barriers and to test the beneficial effect of enlisting pharmacists to transfer knowledge on inappropriate prescriptions simultaneously to both patients and prescribers.

## Methods/Design

### Trial design

#### Study objectives

The primary objective of the trial is to evaluate the effectiveness of a pharmacist-initiated educational knowledge transfer intervention to both patients and prescribers on the discontinuation of inappropriate prescriptions on a community-based sample of chronic inappropriate prescription users as measured by the rate of targeted medication discontinuation at 6 months, with 1-year follow-up to determine whether change rates are sustained over the longterm. The acronym D-PRESCRIBE stands for “*D*eveloping *P*harmacist-led *R*esearch to *E*ducate and *S*ensitize *C*ommunity *R*esidents to the *I*nappropriate prescription *B*urden in the *E*lderly.”

Secondary objectives of the study include: evaluating the added benefit of implicating physicians and pharmacists in a patient-targeted educational intervention on the discontinuation of inappropriate prescriptions in comparison to the EMPOWER [15, 17] study, where patients alone were targeted; to test the transferability of this novel approach to inappropriate prescription discontinuation explored in the EMPOWER study to other classes of inappropriate medications; to better understand the mechanisms by which the educational tool affects participants’ risk perception, knowledge and beliefs with respect to inappropriate prescription use; to evaluate the impact of evidence-based pharmaceutical opinions on physicians’ perception of the prescription as inappropriate; and to document response rates and the overall feasibility of using pharmaceutical opinions as a clinical tool to catalyze physicians to de-prescribe inappropriate prescriptions.

### Design

**Fig. 1:**
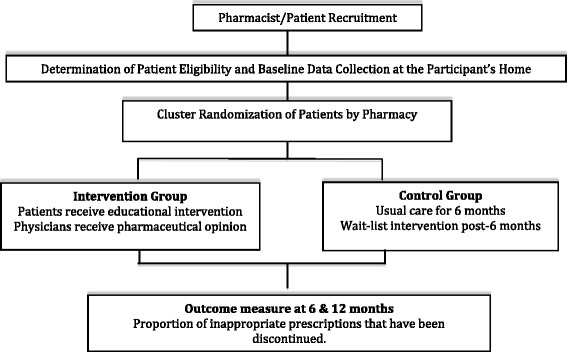
Study flowchart

This is a pragmatic, cluster randomized, parallel-group controlled trial. A cluster design was chosen to prevent contamination across the intervention and control arms by individual clients served by the same pharmacy. The cluster and unit of randomization consists of each community pharmacy. There are two arms in this parallel-randomized controlled trial for each of the four medication categories targeted: the educational intervention arm and the control arm. A 50:50 ratio (intervention: control) of participants will be used in each medication class arm. Figure 1 illustrates the flow chart.

### Study site: clusters and characteristics

The study is being conducted in the greater Montreal area in Quebec, Canada. Collaboration was established with the pharmacies of 3 local drugstore chains within a 2-hour driving radius (approximately100 km) of Montreal. Pharmacies are randomly ordered via a computer-generated program, and subsequently invited to participate in the trial in that order. Clusters consisted of community pharmacies that are able to track medication dispensing, that have a ≥ 20 % older person clientele, and that consent to participate in the project.

### Study population

The study population comprises chronic users of the four targeted classes of inappropriate prescriptions among community-dwelling older adults recruited from community pharmacies in Quebec.

Men and women 65 years of age and older with chronic consumption (>3-month claims) of one of 4 targeted inappropriate prescriptions classes are eligible for participation in this trial. The choice of these 4 medication classes was based on moderate to high quality evidence and the strength of the recommendations presented in the *2012 Updated Beers Guidelines for Inappropriate Prescriptions* [9], as well as their frequency of use in the general population [18–20]. There is a strong recommendation for avoiding the four classes of prescription medications chosen in this trial (see Table 1) with moderate to strong evidence backing these recommendations [9].

**Table 1:** Targeted medication classes

Medication class	Rationale
All benzodiazepines as well as non-benzodiazepine hypnotics	• Associated with:
	○ A 5-fold increased risk of cognitive events [36–39]
	○ A 30 % to 2-fold increased risk of falls [40–42], a 50 % increased risk of hip fractures [42–46]
	○ A 25 % to 2-fold increased risk of motor vehicle accidents [47–49]
	○ Increased risk of Alzheimer’s disease by up to 80 % [50]
	• Similar evidence of harm exists for non-benzodiazepine hypnotics [9]
	• Hypnotics are associated with a greater than 3-fold increased risk of death even when prescribed < 18 pills/year [51]
Anticholinergic agents including first-generation antihistamines (as single agents or as part of combination products)	• Can cause cognitive impairment [39]
	• Have been associated with an increased risk of [52–57]:
	○ Confusion
	○ Dry mouth
	○ Constipation
	○ Functional decline
Long-acting sulfonylurea oral hypoglycemic agents chlorpropamide or glyburide used for the treatment of diabetes	• Estimated to be responsible for 11 % of emergency hospitalizations for adverse drug events in older adults [58]
	• Glyburide is associated with a 52 % greater risk of experiencing at least one episode of hypoglycemia compared with other secretagogues and with 83 % greater risk compared with other sulfonylureas [59, 60]
	• Chlorpropramide has potential to cause SIADH (syndrome of inappropriate antidiuretic hormone secretion) [61]
	• Glyburide was a new addition to the Beers list in 2012 [9, 62]
Chronic non-COX-selective non-steroidal anti-inflammatory drug (NSAIDs)	• Increased risk of gastro-intestinal bleeding/peptic ulcer disease in older adults
	• Ulcers, bleeding, or perforation caused by NSAIDs occur in approximately 1 % of patients treated for 3–6 months, and in about 2–4 % of patients treated for 1 year with trends continuing with longer duration of use [63–65]
	• Use of misoprostol or a proton pump inhibitor reduces this risk, it does not eliminate it

A full list of medication associated with these drug classes is presented in Appendix 1: Table 3

Patients with a diagnosis of severe mental illness or dementia ascertained by the presence of an active prescription for any antipsychotic medication and/or a cholinesterase inhibitor or memantine in the preceding 3 months, those unable to communicate in French and/or English as well as patients showing evidence of significant cognitive impairment (a baseline screening score < 24 on the Mini-Mental State Exam (MMSE) [21]) are excluded. Additionally, patients in assisted-living facilities will be excluded from the study population.

### Ethical approval

The study protocol was approved by the Research Ethics Board of the Centre de Recherche de l’Institut Universitaire de Gériatrie de Montréal, Canada on 17 September 2013 (ClinicalTrials.gov identifier: NCT02053194).

### Enrollment

Enrollment in the trial was conducted in collaboration with three regional pharmacy chains. Company headquarters provided the research team with a list of all chain drugstores with an appropriate version of the pharmacy software within a 100-km radius of the research center. Following this, a high-ranking company representative of each of the three banners circulated an announcement to all pharmacist owners to participate in the project. Following these announcements, pharmacy lists were randomized and then each one contacted systematically in that order to assess interest in participation. Pharmacists interested in participating then met in person with a research coordinator to sign a collaboration engagement, thus confirming their participation in the trial.

### Recruitment of participants and application of eligibility criteria

Participants will be recruited to the trial in a systematic fashion. Participating pharmacists will approve the extraction from the pharmacy software of a comprehensive list of all clients meeting eligibility criteria for the study, divided according to the four targeted drug classes, and listed in random order by drug class. An extraction algorithm was developed and validated to reflect the inclusion and exclusion criteria of participants for the study, and applied across all participating pharmacies. The pharmacist then systematically and sequentially phones each client from each of the four drug classes to invite them to be contacted by the research team for more information about participating in a study on safe medication management, to a maximum of seven consenting participants per drug class or until no more names remain on the lists. The pharmacist records all responses and transfers the names and phone numbers of those who responded affirmatively to the research staff. Research assistants then contact all potential participants referred by the pharmacists (with the client’s permission), re-explains the details to confirm interest in participation and then arrange an appointment at the participant’s residence or at the research center (based on patient preference) to complete the third screening stage: signed consent if eligible and collection of baseline data. During this visit, a research assistant reviews the medication currently taken by the patient, queries the medical history and assesses cognitive function. Signed informed consent to participate in the study is then obtained from individuals who meet the study criteria after baseline cognitive and health status screening. This procedure is followed until three clients from each drug class have been recruited per pharmacy, or until such time as there are no more eligible clients at that pharmacy or clusters have been filled. Participants taking one or more of the targeted drug classes will be randomly assigned to only one group and receive the intervention for a single drug class only.

## Randomization

### Randomization/Concealment of allocation

Randomization will be by pharmacy cluster after recruitment procedures are complete for the cluster. Randomization will be done in blocks using a 1:1 ratio every time to pharmacies and their patients’ complete enrollment and baseline data collection. Allocation of the intervention by a third party will be blinded via a computer-generated random digit generated by a research assistant not involved in participant recruitment, as will data analysis and ascertainment of the outcome. The trial is, nonetheless, considered open-label because both the research assistant who delivers the interventions and the study participants and pharmacists who receive the educational materials will be aware that the intervention is being delivered.

### Blinding

As the intervention is educational in nature, blinding of the intervention is impossible. However, to preserve a certain level of blinding and to protect against sources of bias, the following measures are taken. For participants, blinding is achieved by presenting the project to participants as a project on optimizing medication management. Consenting participants understand that their medication profiles will be transmitted to the research team within the following months and that they will receive a customized letter at some point during the year that may contain recommendations for change, which they can then decide to take to their physician or pharmacist for discussion. For pharmacists, blinding is achieved by presenting the same study timeline. Pharmacists are aware that their clients will receive an intervention at some point during the following year and remain blinded to group allocation throughout the course of the study. Pharmacists also remain blinded to other participating pharmacies. Since pharmacies are randomized as clusters, they are located in distinct geographic locations and generally have no reason to interact with one another. Thus, blinding pertains to both the individual and cluster level.

### Intervention

The intervention is multifaceted, consisting of the delivery of educational materials about inappropriate prescriptions to both patients and their prescribers by the pharmacist. The pharmacist will deliver in person or by mailing the educational material to the patient in the form of a written educational brochure that was developed and tested during the EMPOWER study [15]. All educational material will be customized to the type of inappropriate prescription being consumed by the patient. All materials have already been developed and tested for acceptability [17]. Pharmacists will also provide a letter to their clients explaining why they are receiving an intervention, and a pamphlet inviting them to schedule a consultation. The pharmacist will deliver the educational material to the physician in the form of a faxed pharmaceutical opinion 2 weeks after having delivered the intervention to patients. The research team will provide the pharmacist with the customized educational materials for their patients, and examples of evidence-based pharmaceutical opinions that could be sent to the patient’s physician depending on the type of inappropriate medication consumed. The evidence-based pharmaceutical opinions were developed by the research team, reviewed by experts, field-tested among a cohort of physicians as well as a team of pharmacists, and adapted until consensus was reached on the content and format for the final versions. The evidence-based opinions refer to the Beers criteria and other literature detailing the risk of harm associated with use of each targeted drug class for older adults, and include suggestions for safer therapeutic alternatives. The pharmacist is allowed flexibility in their choice of whether to use the pharmaceutical opinions provided by the research team, adapt it to their needs, draft their own pharmaceutical opinion for the physicians or not send out any opinion at all. All study materials are distributed to each participating pharmacist assigned to the intervention group immediately after randomization.

The comparator for this study will be usual care during the 6-month time period postrandomization. Usual care is a common comparator for a pragmatic trial, since it captures a wide, realistic range of alternate practice scenarios [22]. After enrollment, all pharmacists will be informed that the project materials will be delivered “sometime over the next year.” We will explain to the pharmacists that delays with various study procedures may take 3–6 months and that the recruitment process for the study is long. We will request that no action be taken by the pharmacist other than usual care until such time as the study materials are delivered to them. The control group pharmacists will be given all the educational materials at the end of their 6-month wait period postrandomization.

### Study follow-up

Study follow-ups include 2 telephone calls 1 week and 6 weeks post randomization, and a single in-person interview at 6 months postintervention. Telephone interviews last from 5 to 10 minutes while the final in-person interview may take up to 30 minutes.

### Outcomes

#### Prescription discontinuation rates at 6 months

The primary outcome for the trial is discontinuation of any of the targeted inappropriate prescriptions. The time period for ascertainment of the outcome is 6-months post-intervention. The 6-month time period was selected according to data obtained in the EMPOWER study and is consistent with the transtheoretical model of change, which predicts that once people start thinking about changing their behavior they usually make a decision and implement their plan of action within 6 months [23]. A follow-up at 1 year will be obtained to monitor long-term changes and to assess whether discontinuation persists.

Outcomes will be measured from the administrative database used for public drug claims reimbursement for both the intervention and control groups. This database includes all prescriptions filled at the pharmacy as well payment claims to pharmacists for all services rendered, such as the delivery of pharmaceutical opinions to physicians. Prescription data contain information on all dispensed prescriptions including drug name, dispensation date, dosage, drug form, duration and quantity of the drug dispensed, as well as the license number of the physician who wrote the prescription. Discontinuation of an inappropriate prescription will be defined as the lack of a claims renewal for that medication during a minimum of 3 or more consecutive months (with no subsequent renewals) as well as the absence of initiation of another inappropriate prescription of the same class.

### Secondary outcomes

Medical Research Council guidance for complex intervention studies recommends that process evaluations be conducted within the trial to assess the fidelity and quality of implementation of the intervention, to clarify causal mechanisms, and to identify contextual factors associated with variation in outcomes [24]. We therefore intend to track the sequence of events stemming from the delivery of the knowledge transfer tools to each pharmacist in the intervention group. The following parameters will be measured:

- *Delivery of the educational brochures to the patients* by their pharmacists
- *Prevalence, timing and type of pharmaceutical opinions sent* by the pharmacists to the patients’ primary care providers
- *Effect of the patient knowledge transfer tool* on patients’ beliefs about the use of their inappropriate medications and their intent to discuss cessation with their doctor or pharmacist
- *Effect of the pharmaceutical opinion on the prescriber’s behavior*
- *Patient-physician encounters to discuss inappropriate prescriptions*
- *Patient self-efficacy and improvement in self-efficacy in ability to change medication*

Table 2 illustrates the time points for measurement of each outcome during the study.

**Table 2:** Overview of data collection and measurements in both trial arms

	Baseline	Follow-up		
Visit number	T0	T1	T2	T3
Time	Day 0	7 days post	6 weeks post	6 months post
Inclusion and exclusion criteria	X			
Sociodemographic characteristics	X			
SF-12	X			X
VES-13	X			X
MMSE	X			
PATD	X			X
Blood glucose monitoring		X^c^	X^c^	X^c^
Medication use characteristics	X			
Benzodiazepine Tapering Questionnaire		X^a,b^	X^a,b^	X^a,b^
DTSQs		X^c^		X^c^
Medication risk assessment	X	X		
BMQ-Specific	X	X		
Patient Self-Efficacy Scale	X	X		X
Intervention-related questionnaire		X	X	X
Intervention Appreciation Questionnaire				X

*BMQ-Specific*, Beliefs about Medicines Questionnaire - Specific segment [66]; *DTSQs*, Diabetes Treatment Satisfaction Questionnaire [67]; *MMSE*, Mini-Mental State Exam [68]; *PATD*, Patients Attitude Towards De-prescribing Questionnaire [69]; *SF-12*, 12-Item Short Form Survey to measure health status and health-related quality of life [70]; *VES-13*, Vulnerable Elders Survey [71]. ^a^Only administered if in benzodiazepine group

^b^Only administered if benzodiazepine tapering had begun

^c^Only administered if in sulfonylurea group

### Sample size

The main question driving the sample size is whether the delivery of a knowledge transfer intervention by pharmacists to consumers of inappropriate prescriptions and their prescribers is more likely to result in discontinuation of inappropriate prescription over a 6-month time period compared to usual care. We hypothesize that our intervention will achieve a rate of discontinuation that is at least as great as that achieved in previous studies by medication review by a pharmacist and contact with a physician (maximum rate 27 % in EMPOWER [15]) compared to usual care (maximum rate of discontinuation 6 %) [13, 14, 18, 25–29]. These figures were derived from published studies in older people conducted in the community setting with a non-imposed intervention targeting inappropriate prescriptions, and included a prescription discontinuation measure. We therefore intend to power our study to detect a minimal 20 % increase in any inappropriate medication discontinuation over usual care, and an absolute minimal rate of discontinuation of 25 %, which would compare to EMPOWER. We are also interested in conducting sub-group analyses by drug class as the four drug classes we have chosen have different indications and may have different rates of discontinuation due to the intervention. Our calculations also account for the cluster design, with adjustments made for both clustering and for the effect of the cluster size [30]. We assume that the intracluster correlation coefficient (ICC) will be similar to the ICC observed in the EMPOWER study (0.008) [31]. Based on pilot work from EMPOWER [17], we have chosen the minimal number of participants per drug class (n = 3) in order to augment the likelihood that each consenting pharmacy will achieve the required number of participants. Limiting the number of participants per pharmacy and per drug class should also lower design effects when compared to the EMPOWER study where clusters varied from 2 to 27 participants per pharmacy [30]. With an estimated ICC of 0.05 (worst-case scenario) for the 3 participants recruited per drug class, we would require 17 pharmacies per group (51 participants per arm) to be able to estimate a 20 % absolute discontinuation rate difference between trial arm by drug class with 80 % power and alpha 0.05 [31]. To detect greater differences, a lower sample size is needed. Thus we would have ample power for the overall comparison. Based on preliminary recruitment rates for the D-PRESCRIBE trial during a run-in period, we have observed that only 1 out of every 10 pharmacies that participate are able to recruit the desired number of participants with a participant range per pharmacy of 3–12 and a mean of 6 participants per pharmacy. This may be because smaller pharmacy chains are eligible for inclusion, compared to the EMPOWER trial. Based on our previous research we assume that 10 % of participants will withdraw or be lost to follow-up. We have, therefore, inflated our sample size to 450 participants (112 per medication class) from an estimated 75 pharmacies. Additionally, to compare the added benefit of the pharmaceutical opinion in comparison to the educational material alone, we chose to recruit an additional three participants from the benzodiazepine group. This was powered to detect a minimal 12.5 % difference between participants in this study and the EMPOWER study and accounted for the previously mentioned sample size considerations.

### Analysis

To determine whether randomization was effective, descriptive statistics (means, proportions) will be calculated to assess the balance between the groups on important confounders such as age, sex, health status, baseline beliefs about medications and the degree of polypharmacy. The primary analysis will focus on answering the main research question driving this study - whether the intervention results in an increased discontinuation rate of inappropriate prescriptions of at least 20 % compared to usual care. We will use a marginal model estimated via generalized estimating equations (GEE) with a binary outcome and an identity link, with an exchangeable correlation structure to account for correlation between participants in the same cluster. Participants will be analyzed as randomized (ie, intention to treat). Risk differences between the control and experimental groups will be calculated and the robust variance estimator will be used to estimate the associated 95 % confidence interval and *P*-value [32]. If any confounders (age, sex, degree of polypharmacy or health status) are unbalanced between the groups, we will estimate the unadjusted and adjusted odds ratios for the intervention via a marginal model estimated via GEE with an exchangeable correlation structure. The robust variance estimator will again be used. All analyses described above will be repeated for each drug class during sub-analysis. As a sensitivity analysis, we will compare results obtained with the GEE to other procedures that account for clustering such as generalized linear mixed models.

The fidelity and quality of implementation of the intervention by the pharmacists will be assessed by rates of delivery of the educational materials to the participants and their primary care providers. The types of pharmaceutical opinions delivered and the patients’ and physicians’ responses to receipt of the knowledge transfer tools will be reported as proportions, along with 95 % confidence intervals, and will be stratified by type of prescription. In order to determine whether the patient intervention altered beliefs about the necessity-concern ratio for the inappropriate prescriptions, linear mixed models will be used to evaluate change-scores pre-intervention and postintervention for each medication class with the pharmacist as a random effect. To better understand the explanatory mechanisms driving the success or failure of the intervention, we will track the sequence of events following randomization for each patient in the intervention group. The chronological order of billings for pharmaceutical opinions, prescription changes, and patient visits to the physician for each participant and each type of prescription will be ascertained. These will be compared to the dates and content of the response cards returned by the physicians and the patients’ reports of what transpired during any discussions with health providers about their medication. Analysis of these temporal “pathways” will provide valuable insight into *how* and *why* the de-prescribing process occurred or did not occur for each participant.

## Discussion

The EMPOWER study demonstrated that direct-to-consumer education is effective at eliciting shared decision-making around the overuse of medications that increase the risk of harm in older adults. Our hope here is to demonstrate the added value of using pharmacists as a bidirectional conduit of evidence-based knowledge to patients and physicians to drive the reduction of inappropriate prescriptions. In various countries, legislative and regulatory changes have led to a wider scope of pharmacist practice for substituting or discontinuing certain medications [27]. Data from randomized trials indicate that patients benefit from increased pharmacist involvement in their care [33].

The patient-centered process developed for this study aims to reinforce known enablers and address barriers to medication cessation. By providing the patient with evidence-based information in the educational brochures we expect to increase patient’s endorsement of appropriate cessation, increase their dislike of the medication, reduce the fear of re-emerging symptoms, and equip them with the skills to safely taper their medication. Patient empowerment is a key mechanism for increasing patient responsibility in shared decision-making with health care providers [34]. Use of an evidence-based pharmaceutical opinion aims to catalyze and support pharmacists and physicians by providing them with the appropriate tools and information to positively influence and encourage patients to initiate de-prescribing. Only 41 % of community pharmacists admit familiarity with the Beers criteria of drugs to avoid in older people [35]. As such, the evidence-based pharmaceutical opinion serves a dual purpose in educating both pharmacists and physicians about the latest pharmacogeriatric recommendations. This tripartite educational approach to pharmacists, physicians and patients is intended to achieve synergistic impact.

### Strengths

Strengths of the study include but are not limited to its pragmatic design, which will allow the observed process to reflect real world practice as accurately as possible. Systematic recruitment of participants via community pharmacies, blinding of the study hypothesis from participants, physicians, pharmacists, and evaluators as well as objective assessment of drug discontinuation rates from pharmacy prescription renewal profiles will increase the trial’s internal validity. Comparison with EMPOWER and other studies will allow us to examine the synergic effects of our intervention compared to direct-to-consumer and direct-to-prescriber interventions alone. Additionally, a comparison of discontinuation rates for the four different drug classes may allow us to identify different barriers and/or enablers that need to be addressed for different medication indications.

## Limitations

Limiting the list of inappropriate medications to four drug classes only will restrict the study’s potential generalizability to all inappropriate prescription. Contamination between the experimental and control groups is possible, but we expect it to be minimal. Pharmacists will be informed that the intervention will be staggered over the course of a year and they should follow usual care until receipt of the study materials. Physicians may end up with patients in both the control and experimental arms of the study, but this is unlikely as pharmacies generally serve a specific geographic area and patients will be recruited throughout Quebec. The physician will not be contacted directly because of the potential to influence the outcome of the intervention during the study period and/or to interfere with the pharmacist-doctor relationship. Information on what occurs during the physician-patient encounter will, therefore, be limited.

## Trial status

The trial is currently recruiting participants and is approximately 60 % complete at the time of publication.

## Appendix 1

**Table 3:** Complete list of medications

Benzodiazepines	First generation antihistamines	Long-acting sulfonylurea	Non-COX-selective NSAIDs
Alprazolam	Hydroxyzine	Chlorpropamide	Aspirin (>325 mg/day)
Estazolam	Promethazine	Glyburide	Diclofenac
Lorazepam	Brompheniramine		Diflunisal
Oxazepam	Carbinoxamine		Fenoprofen
Temazepam	Chlorpheniramine		Etodolac
Triazolam	Clemastine		Ibuprofen
Clorazepate	Cyproheptadine		Ketoprofen
Chlordiazepoxide	Dexbrompheniramine		Meclofenamate
Chlordiazepoxide-amitriptyline	Dexchlorpheniramine		Mefenamic acid
Clidinium-chlordiazepoxide	Diphenhydramine (oral)		Meloxicam
Clonazepam	Doxylamine		Naproxen
Diazepam	Triprolidine		Oxaprozin
Flurazepam			Piroxicam
Quazepam			Sulindac
Eszopiclone			Tolmetin
Zolpidem			
Zaleplon			

## Abbreviations

D-PRESCRIBE: Developing Pharmacist-led Research to Educate and Sensitize Community Residents to the Inappropriate prescription Burden in the Elderly
GEE: generalized estimating equations
ICC: intracluster correlation coefficient
MMSE: Mini-Mental State-Exam
NSAIDs: non-steroidal anti-inflammatory drugs

## References

[1] Tannenbaum C (2012). Effect of age, education and health status on community dwelling older men’s health concerns. Aging Male.

[2] Tannenbaum C, Mayo N, Ducharme F (2005). Older women’s health priorities and perceptions of care delivery: results of the WOW health survey. CMAJ.

[3] Reeve E, Wiese MD, Hendrix I (2013). People’s attitudes, beliefs, and experiences regarding polypharmacy and willingness to Deprescribe. J Am Geriatr Soc.

[4] Reason B, Terner M, Moses McKeag A (2012). The impact of polypharmacy on the health of Canadian seniors. Fam Pract.

[5] Terner M, Reason B, McKeag AM (2011). Chronic conditions more than age drive health system use in Canadian seniors. Healthc Q.

[6] Lorgunpai SJ, Grammas M, Lee DS (2014). Potential therapeutic competition in community-living older adults in the U.S.: use of medications that may adversely affect a coexisting condition. PLoS ONE.

[7] Fried TR, O’Leary J, Towle V (2014). Health outcomes associated with polypharmacy in community-dwelling older adults: a systematic review. J Am Geriatr Soc.

[8] Gomez C, Vega-Quiroga S, Bermejo-Pareja F, et al. Polypharmacy in the elderly: a marker of increased risk of mortality in a population-based prospective study (NEDICES). Gerontology. 2014. 10.1159/000365328 25502492

[9] American Geriatrics Society Beers Criteria Update Expert Panel (2012). American Geriatrics Society updated Beers Criteria for potentially inappropriate medication use in older adults. J Am Geriatr Soc.

[10] Gallagher P, Barry P, O’Mahony D (2007). Inappropriate prescribing in the elderly. J Clin Pharm Ther.

[11] McLeod PJ, Huang AR, Tamblyn RM (1997). Defining inappropriate practices in prescribing for elderly people: a national consensus panel. CMAJ.

[12] Information CIfH. In: Information CIfH editor. Drug use among seniors on public drug programs in Canada. Ottawa, ON: CIHI; 2014.

[13] Kaur S, Mitchell G, Vitetta L (2009). Interventions that can reduce inappropriate prescribing in the elderly: a systematic review. Drugs Aging.

[14] Tamblyn R, Eguale T, Buckeridge DL (2012). The effectiveness of a new generation of computerized drug alerts in reducing the risk of injury from drug side effects: a cluster randomized trial. J Am Med Inform Assoc.

[15] Tannenbaum C, Martin P, Tamblyn R (2014). Reduction of inappropriate benzodiazepine prescriptions among older adults through direct patient education: the EMPOWER cluster randomized trial. JAMA Intern Med.

[16] Reeve E, To J, Hendrix I (2013). Patient barriers to and enablers of deprescribing: a systematic review. Drugs Aging.

[17] Martin P, Tamblyn R, Ahmed S (2013). An educational intervention to reduce the use of potentially inappropriate medications among older adults (EMPOWER study): protocol for a cluster randomized trial. Trials.

[18] Monane M, Matthias DM, Nagle BA (1998). Improving prescribing patterns for the elderly through an online drug utilization review intervention: a system linking the physician, pharmacist, and computer. JAMA.

[19] Brekke M, Rognstad S, Straand J (2008). Pharmacologically inappropriate prescriptions for elderly patients in general practice: how common? Baseline data from The Prescription Peer Academic Detailing (Rx-PAD) study. Scand J Prim Health Care.

[20] Médicament Cd. Étude sur la prévalence de l’usage d’ordonnances potentiellement non appropriées (OPNA) chez les aînés du Québec, de 2000 à 2006. In: Médicament Cd, editor. Gouvernement du Québec; 2009.

[21] Cockrell JR, Folstein MF (1988). Mini-Mental State Examination (MMSE). Psychopharmacol Bull.

[22] Zwarenstein M, Treweek S, Gagnier JJ (2008). Improving the reporting of pragmatic trials: an extension of the CONSORT statement. BMJ.

[23] Prochaska JO, Norcross JC, Diclemente CC (1994). Changing for good: the revolutionary program that explains the six stages of change and teaches you how to free yourself from bad habits.

[24] Craig P, Dieppe P, Macintyre S (2008). Developing and evaluating complex interventions: the new Medical Research Council guidance. BMJ.

[25] Stewart R, Niessen WJ, Broer J (2007). General Practitioners reduced benzodiazepine prescriptions in an intervention study: a multilevel application. J Clin Epidemiol.

[26] Gorgels WJ, Oude Voshaar RC, Mol AJ (2005). Discontinuation of long-term benzodiazepine use by sending a letter to users in family practice: a prospective controlled intervention study. Drug Alcohol Depend.

[27] Holden JD, Hughes IM, Tree A (1994). Benzodiazepine prescribing and withdrawal for 3234 patients in 15 general practices. Fam Pract.

[28] Bashir K, King M, Ashworth M (1994). Controlled evaluation of brief intervention by general practitioners to reduce chronic use of benzodiazepines. Br J Gen Pract.

[29] Patterson SM, Hughes C, Kerse N (2012). Interventions to improve the appropriate use of polypharmacy for older people. Cochrane Database Syst Rev (Online).

[30] Eldridge SM, Ashby D, Kerry S (2006). Sample size for cluster randomized trials: effect of coefficient of variation of cluster size and analysis method. Int J Epidemiol.

[31] Dupont WD, Plummer WD (1990). Power and sample size calculations. A review and computer program. Control Clin Trials.

[32] Ukoumunne OC, Forbes AB, Carlin JB (2008). Comparison of the risk difference, risk ratio and odds ratio scales for quantifying the unadjusted intervention effect in cluster randomized trials. Stat Med.

[33] Tannenbaum C, Tsuyuki RT (2013). The expanding scope of pharmacists’ practice: implications for physicians. CMAJ.

[34] Tinetti ME, Basch E (2013). Patients’ responsibility to participate in decision making and research. JAMA.

[35] Zou D, Tannenbaum C (2014). Educational needs, practice patterns and quality indicators to improve geriatric pharmacy care. Can Pharm J.

[36] Glass J, Lanctot KL, Herrmann N (2005). Sedative hypnotics in older people with insomnia: meta-analysis of risks and benefits. BMJ.

[37] Glass JR, Sproule BA, Herrmann N (2003). Acute pharmacological effects of temazepam, diphenhydramine, and valerian in healthy elderly subjects. J Clin Psychopharmacol.

[38] Rummans TA, Davis LJ, Morse RM (1993). Learning and memory impairment in older, detoxified, benzodiazepine-dependent patients. Mayo Clin Proc.

[39] Tannenbaum C, Paquette A, Hilmer S (2012). A systematic review of amnestic and non-amnestic mild cognitive impairment induced by anticholinergic, antihistamine, GABAergic and opioid drugs. Drugs Aging.

[40] Ensrud KE, Blackwell TL, Mangione CM (2002). Central nervous system-active medications and risk for falls in older women. J Am Geriatr Soc.

[41] Landi F, Onder G, Cesari M (2005). Psychotropic medications and risk for falls among community-dwelling frail older people: an observational study. J Gerontol A Biol Sci Med Sci.

[42] Coutinho ES, Fletcher A, Bloch KV (2008). Risk factors for falls with severe fracture in elderly people living in a middle-income country: a case control study. BMC Geriatr.

[43] Tamblyn R, Abrahamowicz M, du Berger R (2005). A 5-year prospective assessment of the risk associated with individual benzodiazepines and doses in new elderly users. J Am Geriatr Soc.

[44] Ray WA, Griffin MR, Downey W (1989). Benzodiazepines of long and short elimination half-life and the risk of hip fracture. JAMA.

[45] Cumming RG, Le Couteur DG (2003). Benzodiazepines and risk of hip fractures in older people: a review of the evidence. CNS Drugs.

[46] Finkle WD, Der JS, Greenland S (2011). Risk of fractures requiring hospitalization after an initial prescription for zolpidem, alprazolam, lorazepam, or diazepam in older adults. J Am Geriatr Soc.

[47] Barbone F, McMahon AD, Davey PG (1998). Association of road-traffic accidents with benzodiazepine use. Lancet.

[48] Thomas RE (1998). Benzodiazepine use and motor vehicle accidents. Systematic review of reported association. Can Fam Physician.

[49] Hemmelgarn B, Suissa S, Huang A (1997). Benzodiazepine use and the risk of motor vehicle crash in the elderly. JAMA.

[50] Billioti De Gage S, Moride Y, Ducruet T (2014). Benzodiazepine use and risk of Alzheimer’s disease: case-control study. BMJ.

[51] Kripke DF, Langer RD, Kline LE (2012). Hypnotics’ association with mortality or cancer: a matched cohort study. BMJ Open.

[52] Agostini JV, Leo-Summers LS, Inouye SK (2001). Cognitive and other adverse effects of diphenhydramine use in hospitalized older patients. Arch Intern Med.

[53] Guaiana G, Barbui C, Cipriani A (2010). Hydroxyzine for generalised anxiety disorder. Cochrane Database Syst Rev (Online).

[54] Han L, McCusker J, Cole M (2001). Use of medications with anticholinergic effect predicts clinical severity of delirium symptoms in older medical inpatients. Arch Intern Med.

[55] Rudolph JL, Salow MJ, Angelini MC (2008). The anticholinergic risk scale and anticholinergic adverse effects in older persons. Arch Intern Med.

[56] Gnjidic D, Bell JS, Hilmer SN (2012). Drug Burden Index associated with function in community-dwelling older people in Finland: a cross-sectional study. Ann Med.

[57] Hilmer SN, Mager DE, Simonsick EM (2009). Drug burden index score and functional decline in older people. Am J Med.

[58] Budnitz DS, Lovegrove MC, Shehab N (2011). Emergency hospitalizations for adverse drug events in older Americans. N Engl J Med.

[59] Gangji AS, Cukierman T, Gerstein HC (2007). A systematic review and meta-analysis of hypoglycemia and cardiovascular events: a comparison of glyburide with other secretagogues and with insulin. Diabetes Care.

[60] Shorr RI, Ray WA, Daugherty JR (1996). Individual sulfonylureas and serious hypoglycemia in older people. J Am Geriatr Soc.

[61] Clarke BF, Campbell IW (1975). Long-term comparative trial of glibenclamide and chlorpropamide in diet-failed, maturity-onset diabetics. Lancet.

[62] Bramlage P, Gitt AK, Binz C (2012). Oral antidiabetic treatment in type-2 diabetes in the elderly: balancing the need for glucose control and the risk of hypoglycemia. Cardiovasc Diabetol.

[63] American Geriatrics Society Panel on Pharmacological Management of Persistent Pain in Older Persons (2009). Pharmacological management of persistent pain in older persons. J Am Geriatr Soc.

[64] Lanas A, Garcia-Rodriguez LA, Arroyo MT (2006). Risk of upper gastrointestinal ulcer bleeding associated with selective cyclo-oxygenase-2 inhibitors, traditional non-aspirin non-steroidal anti-inflammatory drugs, aspirin and combinations. Gut.

[65] Pilotto A, Franceschi M, Leandro G (2003). The risk of upper gastrointestinal bleeding in elderly users of aspirin and other non-steroidal anti-inflammatory drugs: the role of gastroprotective drugs. Aging Clin Exp Res.

[66] Horne R, Weinman J, Hankins M (1999). The Beliefs about Medicines Questionnaire: the development and evaluation of a new method for assessing the cognitive representation of medication. Psychol Health.

[67] Plowright R, Witthaus E, Bradley C. Psychometric evaluation of Diabetes Treatment Satisfaction Questionnaire in 8 languages. Proc Br Psychological Soc. 2000;8(2).

[68] Folstein MF, Folstein SE, McHugh PR (1975). Mini-mental state. A practical method for grading the cognitive state of patients for the clinician. J Psychiatr Res.

[69] Reeve E, Shakib S, Hendrix I (2013). Development and validation of the patients’ attitudes towards deprescribing (PATD) questionnaire. Int J Clin Pharm.

[70] Ware J, Kosinski M, Keller SD (1996). A 12-Item Short-Form Health Survey: construction of scales and preliminary tests of reliability and validity. Med Care.

[71] Saliba D, Elliott M, Rubenstein LZ (2001). The Vulnerable Elders Survey: a tool for identifying vulnerable older people in the community. J Am Geriatr Soc.

